# Adversity in childhood and depression: linked through SIRT1

**DOI:** 10.1038/tp.2015.125

**Published:** 2015-09-01

**Authors:** L Lo Iacono, F Visco-Comandini, A Valzania, M T Viscomi, M Coviello, A Giampà, L Roscini, E Bisicchia, A Siracusano, A Troisi, S Puglisi-Allegra, V Carola

**Affiliations:** 1 Department of Experimental Neurosciences, IRCSS Fondazione Santa Lucia, Rome, Italy; 2 Department of Physiology and Pharmacology, University of Rome ‘La Sapienza,' Rome, Italy; 3 Department of Systems Medicine, University of Rome Tor Vergata, Rome, Italy; 4 Department of Psychology and ‘Daniel Bovet' Center, University of Rome ‘La Sapienza,' Rome, Italy

## Abstract

Experiencing an adverse childhood and parental neglect is a risk factor for depression in the adult population. Patients with a history of traumatic childhood develop a subtype of depression that is characterized by earlier onset, poor treatment response and more severe symptoms. The long-lasting molecular mechanisms that are engaged during early traumatic events and determine the risk for depression are poorly understood. In this study, we altered adult depression-like behavior in mice by applying juvenile isolation stress. We found that this behavioral phenotype was associated with a reduction in the levels of the deacetylase sirtuin1 (SIRT1) in the brain and in peripheral blood mononuclear cells. Notably, peripheral blood mRNA expression of SIRT1 predicted the extent of behavioral despair only when depression-like behavior was induced by juvenile—but not adult—stress, implicating SIRT1 in the regulation of adult behavior at early ages. Consistent with this hypothesis, pharmacological modulation of SIRT1 during juvenile age altered the depression-like behavior in naive mice. We also performed a pilot study in humans, in which the blood levels of SIRT1 correlated significantly with the severity of symptoms in major depression patients, especially in those who received less parental care during childhood. On the basis of these novel findings, we propose the involvement of SIRT1 in the long-term consequences of adverse childhood experiences.

## Introduction

Exposure to traumatic events and receiving little parental care in early life are risk factors for mood disorders in adulthood.^1^ Childhood adversity is associated with significant differences in the clinical picture of depressive illnesses, including earlier onset, a more sustained course of disease, more severe symptoms and worse treatment outcomes.^2, 3, 4^

On the basis of these findings, Teicher and Samson^5^ hypothesized that depressed patients who have experienced childhood maltreatment constitute a distinct clinical ‘ecophenotype' (per Teicher and Samson's terminology) that is likely characterized by specific etiological pathways.^6, 7, 8^ Consistent with this hypothesis, recent studies have shown that depression and inflammation cluster in individuals who have had traumatic childhoods.^9^ Further, downregulation of glucocorticoid-related genes and greater proinflammatory gene expression have been observed in the blood of depressed individuals with early traumatic experiences.^6, 10^

Thus, current research is examining low-grade inflammation as a biological residue of adverse childhood experiences that might affect neurobehavioral changes that lead to depressive symptoms.^11, 12, 13^ However, this hypothesis remains highly debated, and the mechanisms of the long-term maladaptive effects of early stress are unknown.

During early life, epigenetic mechanisms are actively engaged to modify gene expression and function in response to environmental inputs.^14, 15, 16^ Among epigenetic factors, the family of sirtuins (SIRTs, class III histone deacetylases) has garnered interest with regard to long-term modifications because of early stress. SIRTs are NAD^+^-dependent deacylases that act on histones and other substrates to regulate many cellular processes, including aging, inflammation and stress resistance.^17, 18, 19^ SIRTs have many important functions during development, influencing brain structure through axon elongation, neurite outgrowth and dendritic branching.^20^

Further, SIRTs have recently been implicated in mood disorders in mice^21, 22^ and humans,^23^ wherein reduced peripheral blood levels of SIRT1, SIRT2 and SIRT6 have been linked to depressive disorders. However, their function in major depression (MD) is largely unknown, and the contribution of SIRTs to the long-term effects of traumatic childhood has never been examined.

On the basis of the function of SIRTs as regulators of neuronal development and stress responses, we determined the contribution of SIRTs to the progression of depressive disorders following early-life stress. In humans, the diversity of traumatic events and the necessity for a longitudinal approach have created a major obstacle in examining the biological links between an adverse childhood and depression in adulthood, necessitating the use of validated preclinical models.

We applied an environmental manipulation protocol (early social isolation (ESI))^24^ during the third postnatal week to induce depression-like behavior in adult mice. During ESI, juvenile pups simultaneously received less maternal care and had less social contacts with conspecifics. The third postnatal week is characterized by the maturation of several functions—visual, motor and social abilities—that are critical for the interaction of a mouse with its environment.^25, 26, 27^ Moreover, large-scale reconfiguration of the neuronal epigenome and extensive synaptogenesis occur in the mouse brain during this time.^28, 29^ Despite its relevance, the impact of stressful experiences during juvenile age on adult depression-like behavior has not been studied sufficiently.

Using our model, we observed that ESI-induced stress is associated with a significant decline in SIRT1 protein and mRNA in the brain and peripheral blood mononuclear cells (PBMCs) in adulthood, when lower peripheral blood mRNA levels predict greater depression-like behavior. Consistent with these findings, the blood levels of SIRT1 in MD patients correlated with their symptoms and adverse childhood experiences in this group strengthened this relationship. Notably, using a pharmacological strategy, we demonstrated a critical function of SIRT1 during postnatal development in the long-term regulation of depression-like behavior.

## Materials and methods

### Animals

**Figure 1:**
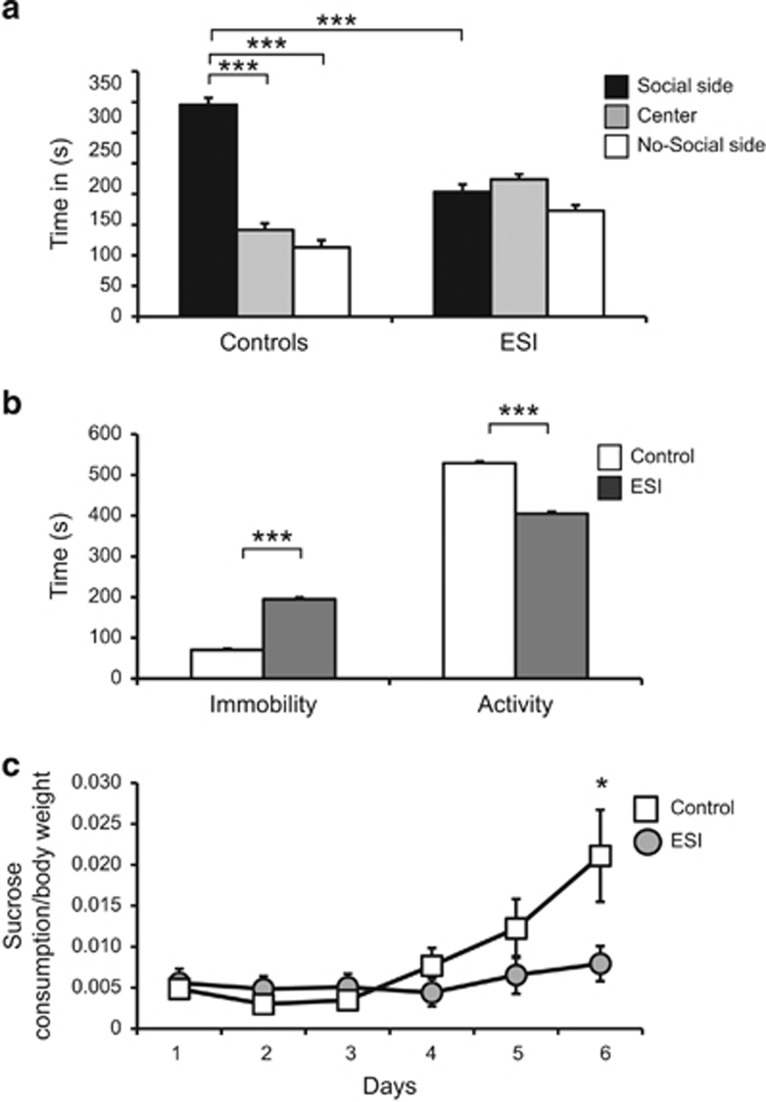
Increased depression-like phenotype in adult early social isolation (ESI)-treated mice. (**a**) Unlike Control mice, adult ESI-treated mice did not prefer the social stimulus (social side) in the social interaction test. (**b**) In the forced swimming test, ESI-treated mice experienced a significant increase in *immobility* and a decline in *activity* compared with control mice. ESI, *n*=18 (male (M)=10, female (F)=8); Control, *n*=16 (M=10, F=6). (**c**) ESI-treated mice did not develop sucrose preference (SP) in the SP test. ESI, *n*=12 (M=8, F=4); Control, *n*=13 (M=7, F=6). ***P<0.001; *P<0.05.

Three-week-old DBA/2J@Ico (DBA), CD 1 (CD1; used for Social stimulus, Figure 1a), C57BL/6J@Ico (C57, used for correlation experiment) male and female mice were purchased from Charles River Laboratories (Calco, Italy). Mice were group-housed on a 12:12 light:dark cycle with lights on at 0700 hours. For the production of pups, DBA mice were mated at 12 weeks of age, and only litters with a number of pups variable between four and eight were included. All procedures were carried out in accordance with European legislation (EEC no. 86/609) and Italian national legislation (DL no. 116/92) governing the use of animals for research. Behavioral, gene/protein expression, correlation and pharmacological experiments were performed in separate groups of animals, resulting in a total number of 133 mice. The experimental sample size was determined with the aid of an online available software (http://www.stat.ubc.ca/~rollin/stats/ssize). Previous experiments that were performed in our laboratory provided the values (for example, means and common s.d.) that are required for the calculation. The investigators were blinded to the group allocation during the experiments and data analysis.

### Social isolation procedure

Mouse pups were randomly assigned to control or ESI group at postnatal day (PD) 14. In the control group mothers and offspring were left undisturbed without cage cleaning until weaning. In the ESI group each pup was singly housed in a novel clean bedding cage for 30 min per day from PD14 to 21. All pups were weaned at PD22 and were tested for either behavioral phenotype or analysis of gene or protein expression at 8–10 weeks of age. Social isolation during adulthood (adult social isolation, ASI) was applied as previously described^30^ by individually housing mice for 20 days at 10 weeks of age.

### Behavioral testing

Mice were exposed to behavioral testing starting at 8 weeks of age (PD60). In order to avoid the effect of prior tests on the mouse behavioral performance, tests were separated by 3-week intervals^31^ and performed in the following order: social isolation test (SIT), forced swimming test (FST) and sucrose preference test (SPT), from the least to the most invasive.

Behavior was recorded and monitored by videotracking (SIT and FST; Ethovision, Noldus Information Technology, Wageningen, the Netherlands) and direct observation (SPT). For the SIRT1-behavior correlation study, the behavior in the FST was evaluated in naive groups of mice (ESI, ASI or C57) 72 h after the blood collection. For the drug-treatment study in early age, the behavior in the FST was evaluated at PD60.

The SIT was carried out according to the method of Sankoorikal *et al.*^32^ Briefly, each mouse was allowed to habituate to the three-chambered plexiglass apparatus containing two-holed cylinders for 10 min. After the habituation, a 4-week-old CD1 stimulus mouse (same sex as the test mouse) was placed in one cylinder and the test mouse could explore the apparatus and the stimulus mouse for the next 10 min. Behavioral data were analyzed by the ‘EthoVision' videotracking system. The ‘time spent' (s) in the three chambers was the parameter used to estimate the mouse preference for the different environments (social versus no social).

The FST was performed as previously described.^33^ Mice were individually introduced in a glass cylinder (25 cm diameter), containing 20 cm of water at 28+2 °C for 10 min, and the amount of *immobility* (absence of movement or small movements of one of the posterior paws that do not produce displacement) and *activity* (vigorous attempts at climbing the walls of the cylinder and active swimming around) was scored for each mouse. Videos were scored blind by highly trained observers (inter-rater reliabilities ⩾0.9; Pearson correlations) using the Ethovision software.

In the SPT, mice were first submitted to 4 days of continuous exposure to water and 5% sucrose solution in their home cage. During the six testing days, each mouse (previously deprived of the sucrose/water bottle for 1 h) was placed in a novel cage equipped with two pipettes (10 ml volume) filled with either water or 5% sucrose solution for 2 h per day. Sucrose consumption per gram of body weight was calculated for each day. The behavioral data were obtained by means of two replications of the experiment.

### Tissue isolation and RNA preparation

For the analysis of brain and PBMC mRNA, mice were killed by decapitation while trunk blood was collected in EDTA tubes. Brains were subsequently dissected, deprived of cerebellum and pons-medulla, and stored at −80 °C. For correlation analysis, blood (200 μl) was instead collected via submandibular puncture. PBMCs were extracted from whole blood using RBC lysis buffer according to the manufacturer's protocol and RNA was subsequently isolated using the Total RNA purification Plus Kit (Norgen Biotek, Thorold, Ontario, Canada). Brain total RNA was isolated using standard Trizol (Invitrogen, Carlsbad, CA, USA) protocol and subsequently purified with DNAse treatment. RNA quantity was determined by absorbance at 260 nm using a NanoDrop UV-VIS spectrophotometer and the quality of RNA was controlled in random samples by running bioanalyzer assays (Agilent Technologies, Palo Alto, CA, USA).

### Quantitative real-time RT-PCR and gene expression analysis

Complementary DNA was obtained using the High Capacity Reverse Transcription Kit (Applied Biosystems, Branchburg, NJ, USA). Complementary DNA templates (10 ng) were processed with quantitative PCR using the 7900HT thermal cycler apparatus equipped with the SDS software version 2.3 (Applied Biosystems) for data collection. Taqman primer sets (Applied Biosystems, see Supplementary Table S1) were used to amplify mouse SIRT1–7, as well as human SIRT1 and *C*_t_ values were normalized to measures of TBP (Tata Binding Protein) and Pgk1 (Phosphoglycerate kinase1) mRNAs for mouse tissues, or Tata Binding Protein and GUSB (Glucuronidase beta) mRNA for human PBMC. All data were run in triplicate and analyzed using the ΔΔ*C*(t) method.^34^

### Histology and immunohistochemistry

Mice were perfused transcardially and brain slices were processed as previously described.^35^ The sections were incubated overnight at 4 °C in phosphate-buffered saline containing 1% bovine serum albumin, 0.3% Triton X-100 as blocking solution, and then in a cocktail of primary antibodies including mouse anti-SIRT1 (NBP1-51641, 1:1000; Novus Biological, Cambridge, UK) and rabbit anti-NeuN (1:400; ABN78, Merck Millipore, Darmstadt, Germany). The specificity of immunohistochemical labeling (SIRT1) was confirmed by omission of primary antibodies and use of normal serum instead (negative controls).

After three washes in phosphate-buffered saline, the sections were incubated in the solution described above for 2 h at room temperature with a cocktail of secondary antibodies including Alexa Fluor 488-conjugated donkey anti-mouse (A-21202) and Alexa Fluor 543-conjugated donkey anti-rabbit (A-31572, 1:200; Invitrogen). In order to avoid staining variability, brain sections of ESI and control mice were concomitantly incubated with the same cocktail of primary and secondary antibodies. Sections were rinsed, 4,6-diamidino-2-phenylindole-counterstained, mounted, coverslipped and then examined using a confocal laser scanning microscope (Zeiss LSM700, Oberkochen, Germany). The confocal image acquisitions were performed using consistent settings for laser power and detector gain.

### Densitometric analyses of fluorescence images

Quantification of the SIRT1 immunoreactivity in the different brain regions (motor cortex, striatum, dentate gyrus and basolateral complex of the amygdala) was performed by densitometric analysis. All quantitative analyses were conducted blind to the animal's experimental group. After confocal acquisition, images were exported in TIFF and analyzed with the ImageJ software (http://rsb.info.nih.gov/ij/; National Institutes of Health, Bethesda, MD, USA).

The background signal was determined in a non-stained area. The threshold was adjusted according to the background signal and kept constant between sections. SIRT1-associated signal was quantified by manually outlining the areas of interest. For striatum, the mean signal intensity (*F*) of SIRT1 was performed on two squared frames (100 μm per side) pseudorandomly distributed dorsoventrally on six sections sampled to cover the rostrocaudal extent of the striatum (12 samples per mouse). For motor cortex (M1), the *F* of SIRT1 was performed on two squared frames (100 μm per side) pseudorandomly distributed mediolaterally on six sections sampled to cover the M1 rostrocaudal extent entirely (*n*=12 samples per mouse). For the dentate gyrus, the *F* of SIRT1 was performed on one squared frame pseudorandomly distributed mediolaterally on 12 sections sampled to cover the rostrocaudal extent of the area. Finally, for the basolateral complex of the amygdala mean signal intensity (F) of SIRT1 was performed on one squared frame per section, always distributed at the same position, on six sections sampled to cover the rostrocaudal extent of the nucleus. The F/A ratio defines the mean fluorescence of individual samples (F) normalized to total cellular surface (A).^35^ Accordingly, quantification was performed on five mice per group.

### Drugs and pharmacological treatments

Selisistat (EX-527, Selleckchem, Munich, Germany, S1541) and Resveratrol (Sigma, St Louis, MO, USA) were used for SIRT1 inhibition and activation, respectively. Selisistat (6-chloro-2,3,4,9-tetrahydro-1H-carbazole-1-carboxamide) is one of few SIRT1 inhibitor compounds for which mechanistic data are available^36^ and that combine high potency with significant isoform selectivity.^37^ It inhibits SIRT1 with an IC50 value of 0.1 μM, ∼100-fold more potently than SIRT2 and Sirt3 and has no effect on SIRT5′s deacetylation activity.^36, 38^ The compound has low clearance and complete oral bioavailability as well as a 2:1 brain:plasma ratio in mice and absence of overt toxicity in the mouse at dose levels up to 100 mg kg^−1^.^39^ The chronic administration (8 weeks of treatment) of selisistat at the oral dose of 20 mg kg^−1^ has been recently demonstrated to be effective in improving survival in the R6/2 transgenic mouse model of Huntigton disease. No previous research has ever employed selisistat in young mice.

Resveratrol (trans-3,4′,5-trihydroxystilbene) is a natural polyphenolic compound shown to significantly increase SIRT1 activity through an allosteric interaction, resulting in the increase in SIRT1 affinity for both NAD+ and the acetylated substrate.^40^ Together with SIRT1, Resveratrol has been shown to modulate a number of targets, including Cyclooxygenase 1 and 2, cAMP-specific phosphodiesterase (phosphodiesterases 1, 3 and 4), PPARγ and δ, Phosphokinases (PKCα, βI and PKD1).^41^ The specificity of SIRT1 activation by Resveratrol has been recently debated; however, its oral bioavailability, permeability to the brain–blood barrier and commercial availability justify its common use for the experimental activation of SIRT1.^42^ Control and ESI-treated mice received daily oral gavage (10 ml kg^−1^) of resveratrol (50 mg kg^−1^), selisistat (EX-527, Selleckchem, S1541, 20 mg kg^−1^) or vehicle (0.5% hydroxylpropylmethyl cellulose, Sigma, in sterile water) from PD14 to PD25. Suspensions were prepared weekly and aliquoted for daily dosing. Vigorous agitation of the vial preceded each administration. For ESI-treated mice, drug was administered 30 min before the stress application.

### Human studies

The clinical sample referred to the psychiatric clinic at the University of Rome ‘Tor Vergata' and consisted of 27 patients having received a DSM-IV-TR diagnosis of MD, under current depressive state. Control group included 19 healthy volunteers recruited among researchers of the IRCSS Fondazione Santa Lucia (Supplementary Table S2). Inclusion criteria were as follows: age over 30 years old and history of depression (more than two episodes throughout their life) for MD patients or absence of current or past psychiatric disorders for controls, as confirmed by diagnostic interview. Biomarkers of inflammation were not assessed in the two groups. However, none of the participants had a clinical diagnosis of chronic inflammatory disease (for example, celiac disease, vasculitis, lupus, chronic obstructive pulmonary disease, irritable bowel disease, arthritis or psoriasis).

Before clinical assessment, all participants were given a complete description of the study. All data were obtained under informed consent and using procedures approved by the University of Rome ‘Tor Vergata' Intramural Ethics Committee and Fondazione Santa Lucia Ethics Committee. The experimental sample size was determined with the aid of the online available software (http://www.stat.ubc.ca/~rollin/stats/ssize). To perform this calculation, we used values (for example, means and common s.d.) obtained from similar experiments previously performed in the laboratory of our collaborator (AT).

### Clinical assessment

The severity of depressive symptoms was measured with the Beck Depression Inventory (BDI) and the 17-item Hamilton Depression Rating Scale (HAMD). The combined use of a clinician-rating scale (HAMD) with a self-rating scale (BDI) allowed us to integrate psychiatrist's assessment of the intensity of depression with patients' evaluation of their emotional distress.^43^ However, the BDI scale was used to decide whether the patient was in remission (BDI total score <10) or not (BDI total score >10). Cutoff scores were applied as previously described.^44^

Furthermore, parental care experienced in childhood was measured using the Parental Bonding Inventory (PBI).^45, 46^ The questionnaire is retrospective, meaning that adults (over 16 years) complete the measure for how they remember their parents during their first 16 years. The PBI includes two subscales assessing maternal and paternal care. These scales consist of items querying the quality of subjects' relationship with their parents during childhood (for example, ‘My mother spoke to me in a warm and friendly voice'). Participants report on a four-point scale how true each statement was of their own experiences. The participants of this study were assigned to low-care or high-care groups on the basis of their maternal and paternal care scores, using the suggested cutoff scores by Parker and Lipscombe.^45^ Patients who reported scores lower than 27 on PBI maternal care scale and 24 on PBI paternal care scale were classified as low-care patients, whereas the others were considered high-care patients (Supplementary Table S3). The requirement of both maternal and parental care lower than cutoff in the low-care group was chosen in order to include only patients with severe lack of care.

### Human RNA

Blood (5 ml) was drawn on all participants, collected in EDTA vacutainer (BD, Toronto, Ontario, Canada), and PBMC and mRNA were isolated within few hours from collection as described for mouse studies.

### Statistics

All data obtained were initially checked for homogeneity of variance, with measures failing Levene's test analyzed by nonparametric Mann–Whitney procedures. In the mouse study, all other parameters were subjected to parametric either two-way analysis of variance (analysis of variance; SIT, FST, FST after pharmacological treatments and SIRT1–7 RNAs) or repeated-measure analysis of variance (SPT). Analysis of variance was followed, in cases of significance (*P*<0.05), by *post hoc* comparisons using Duncan's test. In the human study, comparisons between groups were performed using the *χ*^2^-test on categorical measure (Gender ratio). Parametric Student's *t*-test was used to analyze continuous measures (Age, SIRT1 RNA, BDI and HAMD scores). Regression and Pearson correlation analyses were performed to evaluate the correlation between SIRT1 expression levels and immobility in the FST/depressive symptoms. Statistical analyses were carried out with the help of Statistica software Version 12.0 (StatSoft, Tulsa, OK, USA).

## Results

### ESI increases depression-like behavior in adult mice

As in humans, the quality of the early postnatal environment shapes adult behaviors in rodents.^24^ To test the possibility that juvenile stress has an impact on adult behavior in mice, we administered ESI stress to DBA mouse pups during the third postnatal week and evaluated the adult behavioral phenotype. Three behavioral tests—social interaction, forced swimming and sucrose consumption—were used to measure various components of depression-like behavior—that is, social phobia, hopelessness and anhedonia, respectively.

In the SIT, ESI-treated mice did not show any preference for the social compartment, whereas control mice spent significantly more time in the social chamber (Figure 1a; time x environment: F[2,60]=14.00, *P*=0.001). In the absence of the stimulus mouse, no preference for compartments was noted in either group (Supplementary Figure S1). In FST, ESI-treated mice experienced a significant increase in *immobility* and a decline in *activity* (Figure 1b; environment: F[1,30]=49.58, *P*<0.001 and F[1,30]=49.70, *P*<0.001) behaviors compared with control mice. In the SPT, ESI-treated mice did not develop a preference for the sucrose solution (Figure 1c, Supplementary Figure S2; time x environment: F[5,105]=3.59, *P*=0.005).

No gender effect was observed (SIT, gender: F[1,30]=0.05, P=0.829; FST *immobility,* gender: F[1,30]=0.04, P=0.852; FST *activity,* gender: F[1,30]=0.04, *P*=0.842; SPT, gender: F[1,21]=1.58, *P*=0.223).

### ESI induces long-term changes in sirtuins mRNA expression in the mouse brain and PBMCs

**Figure 2:**
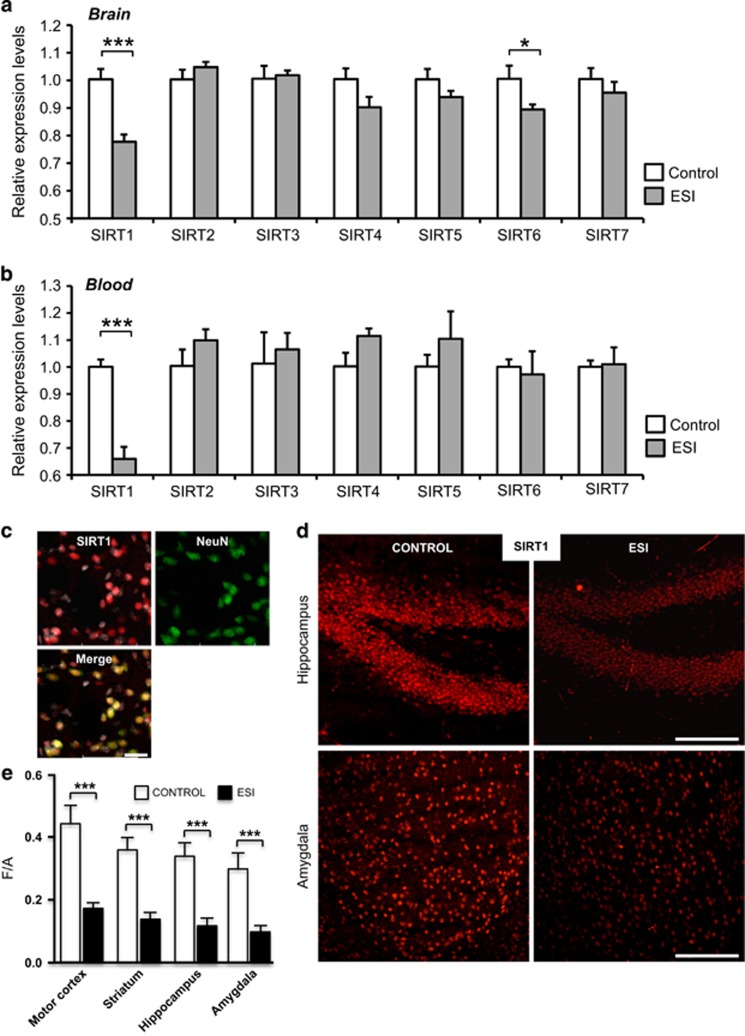
Early social isolation (ESI) induces long-term changes in sirtuin1 (SIRT) mRNA expression in the mouse brain and blood and reduces the SIRT1 protein level in different brain regions. (**a**) The mRNA expression levels (relative expression levels=fold changes over control, normalized to *PGK1* and *TBP* (Tata Binding Protein) genes) of SIRT1 and SIRT6 were significantly decreased in the brain of ESI compared with control mice. (**b**) In peripheral blood mononuclear cells (PBMCs) of ESI-treated mice, the expression level of SIRT1 mRNA was downregulated. ESI, *n*=9 (male (M)=6, female (F)=3); Control, *n*=7 (M=4, F=3) ***P<0.001; *P<0.05. (**c**) Confocal images showing SIRT1 (red) and NeuN (green) immunostaining plus 4,6-diamidino-2-phenylindole (DAPI) counterstaining (gray) in the brain of control mice. SIRT1 (red) colocalizes both with the neuronal marker NeuN (green) and with DAPI (gray). (**d**) Confocal images from the hippocampus and basolateral amygdala of control and ESI mice reacted with SIRT1 antibody. (**e**) Densitometric analysis of SIRT1 immunoreactivity in the motor cortex, striatum, hippocampus and basolateral amygdala revealed a significant reduction of SIRT1 immunoreactivity in ESI compared with control mice. The F/A ratio defines the mean fluorescence of individual samples (F) normalized to total surface (A). ESI, *n*=5; Control, *n*=5. ***P<0.001. Scale bars: (**d**) 100 μm; 25 μm.

Increasing evidence has demonstrated the involvement of SIRTs in the regulation of mood disorders. Recent human studies have shown that a reduction in peripheral SIRT1, 2 and 6 expressions is associated with depressive disorders.^23^ These findings prompted us to measure the mRNA levels of SIRTs (SIRT1–7) in the brain and in PBMCs of adult ESI-treated and control mice (Figure 2a and b). We observed significantly less SIRT1 and SIRT6 mRNA in total brain lysates of ESI-treated versus control mice (Figure 2a; environment: F[1,12]=20.59, *P*=0.001 and F[1,12]=4.80, *P*=0.049).

Consistent with previous findings and our results in the brain, SIRT1 mRNA levels were downregulated in PBMCs of ESI-treated versus control mice (Figure 2b; environment: F[1,12]=42.78, *P*=0.003). When we corrected for multiple comparisons, the significant difference in SIRT1 expression in the brain and PBMCs remained between ESI-treated and control animals.

No gender effect was observed (SIRT1, gender: F[1,12]=0.272, *P*=0.6118; and SIRT6, gender: F[1,12]=1.09, *P*=0.318; blood SIRT1, gender: F[1,12]=0.125, *P*=0.729).

### ESI effects a long-term reduction in SIRT1 protein levels in the mouse brain

Our findings suggested that lower SIRT1 content is a long-lasting biological residue of ESI-induced stress. We hypothesized that if SIRT1 mediates the behavioral alterations in ESI-treated mice, then the resulting transcriptional changes should reflect a decrease in SIRT1 protein levels in the brain. Thus, we analyzed the brains of adult ESI-treated and control mice using confocal microscopy to measure the differences in SIRT1 protein level and distribution.

Consistent with the mRNA data, using densitometric analysis of SIRT1 immunostaining, SIRT1 was downregulated in ESI-treated versus control mice in various regions of the brain, including the motor cortex, striatum, hippocampus and amygdala (Figure 2c and e; *U*=0.00, *P*<0.001, *r*=0.86; *U*=31.00, *P*<0.001, *r*=0.84; *U*=0.00, *P*<0.001, *r*=0.86; *U*=25.00, *P*<0.001, *r*=0.84).

### SIRT1 mRNA expression in PBMCs correlates with despair-like behavior in ESI mice

The behavioral activity in the FST was widely distributed in ESI-treated mice compared with control animals, as shown by the frequency distribution histograms for *immobility* (Supplementary Figure S3). This finding suggests that ESI-treated mice embed a disparate susceptibility to the ‘adverse' juvenile experience, which is in turn translated into different levels of behavioral ‘despair' thus, we hypothesized that a reduction in SIRT1 linked the susceptibility to juvenile stress. If this model was true, we would expect variations in SIRT1 levels to correlate significantly with depression-related measures in ESI-treated mice but not in animals in which a depression-like phenotype was induced by similar isolation stress during adulthood.

**Figure 3:**
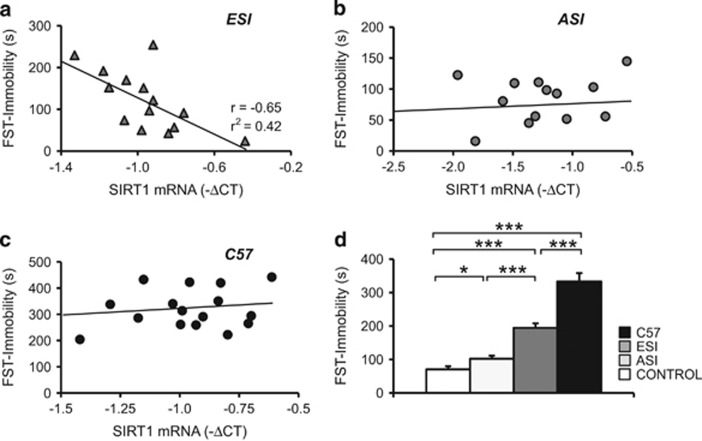
Blood sirtuin1 (SIRT1) mRNA expression correlates with the behavioral ‘despair' in early social isolation (ESI) but not in adult social isolated (ASI) or C57 mice. (**a**) Regression analyses revealed significant correlation between mRNA SIRT1 expression (inverse of Δ*C*_t_) measured 2 days before the behavioral test and forced swimming test (FST) *immobility* in ESI mice. (**b**) Correlation was absent in mice stressed during adulthood (ASI) and (**c**) in C57 mice. (**d**) ESI, ASI and C57 mice experienced higher *immobility* compared with control mice. ESI, *n*=14 (male (M)=6, female (F)=8); ASI, *n*=15 (M=6, F=9); C57, *n*=16 (M=8, F=8) and Control, *n*=10 (M=7, F=6). ***P<0.001; *P<0.05.

To test this possibility, based on the peripheral changes in SIRT1, we first measured SIRT1 mRNA levels in PBMCs in a new group of ESI-treated mice several days before their behavioral performance in the FST was evaluated. We noted a significant negative correlation between peripheral SIRT1 mRNA expression and the level of *immobility* in the FST, meaning that lower SIRT1 levels are associated with a more extensive depression-like phenotype in ESI-treated mice (Figure 3a; *r*=0.65, *P*<0.001). No significant link was observed between SIRT1 expression and *immobility* in the FST in a model of ASI-induced depression^30^ (Figure 3b; *r*=−0.24, not significant).

To exclude the possibility that the downregulation in SIRT1 is directly associated with depressive-like behavior—independent of a stress response—we performed Pearson correlation analysis between SIRT1 mRNA levels in PBMCs and *immobility* in the FST in a naive inbred mouse line (C57) that shows high levels of behavioral despair in the FST.^33^ Again, no correlation was found between these parameters (Figure 3c; *r*=−0.22, not significant). ESI-treated, ASI-treated and C57 mice showed increased levels of *immobility* compared with control mice (Figure 3d; environment: F[3,56]=48.84, *P*<0.001; gender: F[3,56]=0.05, *P*=0.832).

### Pharmacological modulation of SIRT1 during postnatal development alters depression-like behavior in adult mice

Our findings indicate that downregulation of SIRT1 correlates in the long term with the susceptibility to experiencing depression following early-life adversities. To determine the contribution of SIRT1 to the development of depression, we pharmacologically activated or inhibited SIRT1 function with resveratrol and selisistat (EX-527), respectively, during juvenile age and measured the effects on depression-related behavior in adulthood. We administered vehicle, resveratrol (50 mg kg^−1^) or selisistat (20 mg kg^−1^) to control and ESI-treated mice by daily oral gavage from PD14 to PD25 and measured the levels of behavioral despair in adulthood.

**Figure 4:**
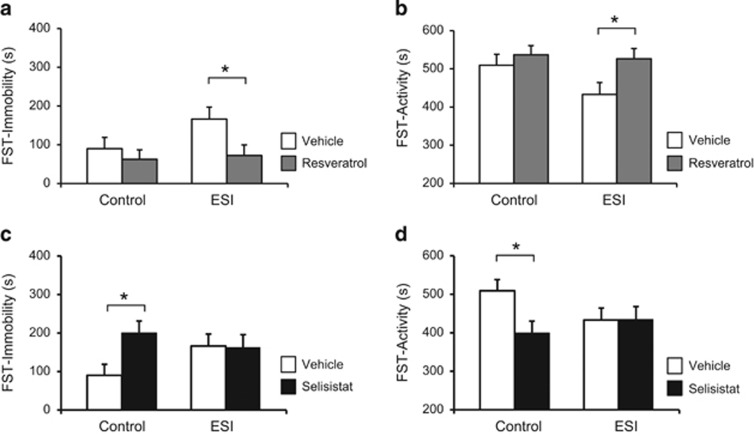
Pharmacological modulation of sirtuin1 (SIRT1) during juvenility alters despair-like behavior in adult mice. (**a**) Adult early social isolation (ESI) mice treated with resveratrol (50 mg kg^−1^) from PD14 to PD25 display decreased *immobility* and (**b**) increased *activity* in the forced swimming test (FST). Resveratrol treatment did not alter the FST performances in Control mice. (**c**) Adult control mice treated with the selective SIRT1 inhibitor selisistat (20 mg kg^−1^) from PD14 to PD25 display increased *immobility* and (**d**) decreased *activity* in the FST. Selisistat treatment did not alter the FST performances in ESI mice. Control: vehicle, *n*=12 (M=8, F=4); resveratrol, *n*=7 (M=2, F=5); selisistat, *n*=6 (M=2, F=4). ESI: vehicle, *n*=9 (M=4, F=2); resveratrol, *n*=8 (M=3, F=5); selisistat, *n*=7 (M=3, F=4). *P<0.05.

Whereas resveratrol had no significant effect on control mice in the FST, it reduced *immobility* (Figure 4a; environment x treatment: F[1,22]=4.52, *P*=0.049) and increased *activity* (Figure 4b; environment x treatment: F[1,22]=4.53, *P*=0.049) in ESI-treated animals compared with vehicle. In contrast, control mice that were given selisistat showed significantly greater *immobility* (Figure 4c; environment x treatment: F[1,20]=5.05, *P*=0.036) and lower *activity* (Figure 4d; environment x treatment: F[1,20]=5.05, *P*=0.036) in the FST versus vehicle. These behavioral changes mirrored the increased behavioral despair in vehicle-treated ESI mice. Moreover, selisistat treatment in ESI mice failed to alter *immobility* in the FST compared with vehicle.

Similar chronic treatment (10 days) with selisistat or resveratrol in adulthood did not induce changes in depression-like behavior (Supplementary Figure S4A and B; selisistat *immobility,* treatment F[1,9]=0.59, *P*=0.470; resveratrol *immobility,* time x treatment: F[1,8]=0.36, *P*=0.560) or locomotion (Supplementary Figure S5; resveratrol *total locomotion*, *U*=8.00, *P*=0.710, *r*=0.122). No gender effect was observed (selisistat *immobility*, gender: F[1,20]=2.14, *P*=0.158; resveratrol *immobility*, gender: F[1,22]=0.01, *P*=0.916). Overall, these findings are consistent with SIRT1 having a critical function in establishing the ESI-induced depression-like phenotype.

### SIRT1 mRNA expression in PBMCs correlates with the severity of depressive symptoms in humans

**Figure 5:**
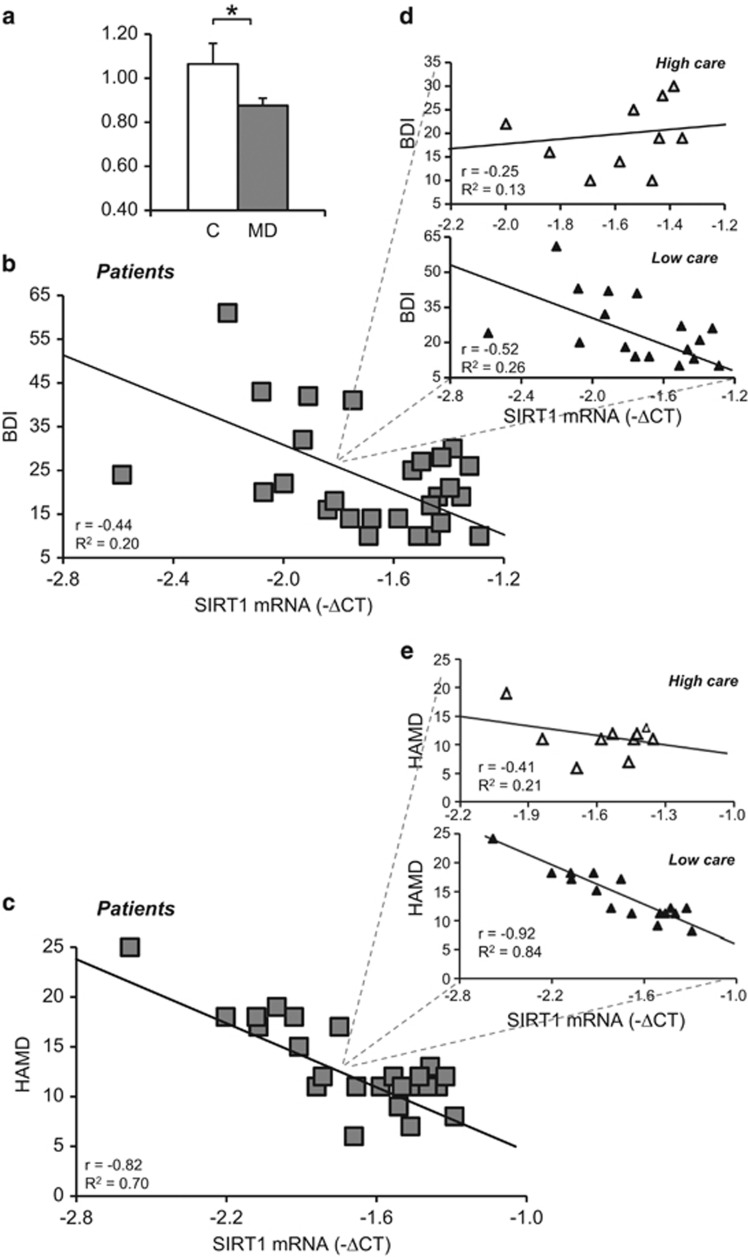
Blood sirtuin1 (SIRT1) level is decreased in major depression (MD) patients. (**a**) MD patients had significantly lower SIRT 1 mRNA level (relative expression levels=fold changes over control, normalized to *PGK1* and *GUSB* genes) than control subjects. (**b**, **c**) Significant correlations between SIRT1 level of expression (inverse of Δ*C*_t_), Beck Depression Inventory (BDI) and HAMD scores were observed in MD patients. (**d**, **e**) When the clinical population was divided into ‘low-care' and ‘high-care' groups—based on history of parental care—highly significant correlations were observed only in the ‘low-care' group. Control, *n*=19 (M=6, F=13); MD patients, *n*=27 (M=6, F=21). High care, *n*=10; low care, *n*=17. *P<0.05. HAMD, Hamilton Depression Rating Scale.

On the basis of our results, we designed a pilot study to determine whether SIRT1 is modulated in a clinical population. We measured SIRT1 transcript levels in the PBMCs of MD patients in a current depressive state and healthy control subjects (Supplementary Table S2). We first determined whether SIRT1 was a marker of depression; as per this hypothesis, patients had significantly lower SIRT1 levels than control subjects (Figure 5a; clinical state: *t*[44]=2.15, *P*=0.037).

We then tested whether SIRT1 expression in PBMCs correlated with depressive symptoms. We noted a significant correlation between SIRT1 expression and BDI (Figure 5b; *r*=0.45; *R*^2^=0.197, *P*=0.040) and HAMD scores (Figure 5c; *r*=0.82; *R*^2^=0.700, *P*<0.001) in MD patients. SIRT1 mRNA expression and BDI scores were not associated in healthy controls (Supplementary Figure S6). Then, we examined whether the quality of the environment during childhood contributed to this relationship. The selection of low-care patients in the entire clinical sample strengthened the correlation between SIRT1 mRNA expression and the severity of depressive symptoms (Figure 5d and e; BDI, *r*=0.52; *R*^2^=0.263, *P*=0.045; HAMD *r*=0.92; *R*^2^=0.840, *P*<0.001), whereas in high-care patients, this connection was absent (Figure 5d and e; BDI *r*=−0.25; *R*^2^=0.125, *P*>0.050; HAMD *r*=0.41; *R*^2^=0.205, *P*>0.050). These results also implicate SIRT1 in MD—specifically when associated with an adverse childhood.

## Discussion

The link between adverse childhood and depression in adulthood has been widely described.^1^ In preclinical models, environmental manipulation at an early age alters adult behavioral phenotype.^24, 47^ Research in this field has concentrated primarily on modulating maternal care during the first 2 weeks of life.^48, 49, 50^ Nevertheless, the third postnatal week is a critical period in the development of adult behavior.^51^

Our findings show that exposure of mice to social stress during the third postnatal week increases depression-like behavior in adulthood, characterized by lower sociability, greater despair behavior and anhedonia. In the ESI protocol, social contacts are limited during a particularly sensitive developmental time, in which the maturation of visual and motor abilities accompanies the initial elements of sociability among siblings.^25, 26, 27^ These new environmental inputs are translated into long-lasting changes in brain function, attributed to strong neuronal plasticity, extensive synaptogenesis and active epigenetic processes,^28, 29, 52^ all of which cooperate during this age. As a result, we reason that mild environmental interference during physiological growth, such as ESI, affects chronic behavioral and biochemical changes.

The advantage of preclinical models is that they allow one to perform parallel molecular studies in the brain and blood. The ESI model permits us to observe the downregulation of SIRT1 mRNA in mouse PBMCs, similar to what has been seen in depressive patients.^23^ Moreover, it extends our investigation to the brain, in which we noted similar alterations in SIRT1 mRNA and protein. Recent studies have shown that electroconvulsive shock, an antidepressant treatment, increases SIRT1 immunoreactivity in the mouse hippocampus and hypothalamus,^22^ whereas social defeat downregulates SIRT1 in the ventral hippocampus in rats.^53^

These findings are consistent, with SIRT1 activity in the brain being lower in depression-like behavior; however, the function of SIRT1 in mood disorders remains unknown. Libert *et al.*^21^ reported that mice that lacked the SIRT1 gene were more resistant to the depression-like phenotype than their wild-type littermates in the FST and in social defeat-induced sucrose anhedonia. This phenotype was associated with a decrease in monoamine oxidase A expression in SIRT1 KO mice—in contrast to our findings. Nevertheless, the absence of the SIRT1 gene from conception to throughout life, as in a genetic deletion model, can hardly be compared with physiological modulation of SIRT1 protein, as in ESI-treated mice. In our study, the decrease in SIRT1 by ESI-induced stress was insufficient to alter monoamine oxidase A expression in the blood and brain of adult ESI mice (data not shown). Thus, we hypothesize that in the ESI model, SIRT1 changes specific mechanisms during postnatal development that regulate the depression-like phenotype. The absence of an effect of SIRT1 modulation on depression-like behavior in adults is consistent with this hypothesis. The extensive epigenomic remodeling that occurs in the third week of life in mice^28^ implicates SIRT1 histone deacylase activity and its function in epigenetic regulation^54^ as critical mechanisms that underlie its long-lasting impact on behavioral phenotypes.

This hypothesis is consistent with the results of our correlation experiments, in which peripheral SIRT1 mRNA levels in adult ESI mice predicted the level of behavioral despair in the FST, thus linking SIRT1 downregulation to the susceptibility to ESI-induced stress. The lack of a correlation between SIRT1 levels and performance in the FST in mice that experienced similar isolation stress in adulthood (ASI) or those with a naturally high level of depression-like phenotype (C57) strengthens the hypothesis that only stress at an early age interferes with SIRT1 function and confirms that in mice, similar to what happens in humans, distinct etiological pathways characterize the early stress-induced depression-like phenotype.

As in our mouse study and consistent with the findings of Abe *et al.*,^23^ we found that SIRT1 mRNA expression in PBMCs declined in MD patients, correlating negatively with the severity of symptoms. Notably, early adverse experiences influenced the relationship between SIRT1 mRNA levels and symptom severity, again suggesting that SIRT1 is linked to the pathological symptoms and that it mediates the pathogenesis that is triggered by early adversity. The translation of these findings from mouse to humans has improved the construct validity of the ESI model. Conversely, our study raises the possibility of using SIRT1 as a peripheral biomarker in diagnosing and stratifying MD patients.^55^ Nevertheless, our clinical data should be interpreted in light of the low number of subjects in the two groups, necessitating replication of this study in larger samples.

Our pharmacological experiment was critical to demonstrate the involvement of SIRT1 in the ESI-induced stress response and regulation of adult behavior. However, some limitations of this study should be acknowledged: first, whereas SIRT1 activation by resveratrol during ESI-induced stress was sufficient to prevent behavioral alterations in adults, resveratrol effects on SIRT1 may be indirect; thus, other substrates may have been involved (see Materials and methods section). For example, through the inhibition of cAMP-specific phosphodiesterase, resveratrol activates cAMP signaling pathway, which then leads to activation of SIRT1.^56^ In the future, this study could be implemented with a more specific approach such as viral-mediated overexpression of SIRT1 for the identification of critical brain areas involved. Second, we showed that the selective inhibition of SIRT1 via selisistat in juvenile control mice exacerbated adult behavioral despair in the FST. However, we cannot exclude that this effect on immobility could be dependent of changes in general locomotor activity induced by the drug. To address this issue, the effects of this treatment on other aspects of the depression-like behavior independent from locomotion (for example, sucrose consumption) should be investigated in the future.

SIRT1 function in maladaptive responses to traumatic childhoods has never been described. Through its NAD-dependent deacylase activity, SIRT1 governs several genetic programs to cope with changes in the cell status (for example, inflammatory, metabolic or oxidative stress), generally orchestrating prosurvival mechanisms.^57, 58, 59, 60, 61^ Its protective function has been described extensively in aging and cardiovascular disease,^62, 63^ and a growing body of evidence is demonstrating many important functions of SIRT1 in regulating brain development, through axonal elongation, neurite outgrowth and dendritic branching.^64, 65, 66^ We hypothesize that the ESI-induced stress, by interfering with normal SIRT1 expression and function, affects neurobehavioral adjustments during postnatal development that manifest in the long term as a depression-like phenotype.

We propose that SIRT1 is a developmental component of the response to childhood adversity that increases the susceptibility to depression. Future studies should evaluate the responsiveness of ESI-treated mice to antidepressants and examine specific treatment responses on the depression-like phenotype that is associated with early-life adversities. Moreover, to determine the contribution of SIRT1 activity in the pathophysiology of early stress-associated depression, the ‘developmental dynamics' of ESI-induced changes in SIRT1 expression throughout life in mice must be followed, and SIRT1-dependent alterations in biological mechanisms during ESI should be identified, guiding the identification of pharmacological targets for preventive and therapeutic interventions.

Increasing evidence is implicating low-grade inflammation in childhood trauma-associated depression.^9, 10, 11, 12^ Considering the anti-inflammatory function of SIRT1,^58, 59^ examining the crosstalk between SIRT1 and inflammation in the ESI model of depression would advance our understanding of these mechanisms. Moreover, recent studies have observed that traumatic childhood and depression often forecast one's vulnerability to such conditions as cardiovascular and aging diseases.^67, 68^ The protective function of SIRT1 in this context should improve our understanding of the underlying mechanisms.
