# Supplementary material for: Adversity in childhood and depression: linked through SIRT1
Source: Transl Psychiatry. 2015 Sep 1;5(9):e629–. doi: 10.1038/tp.2015.125 (PMC5068813; doi:10.1038/tp.2015.125)
Supplement: Supplementary Information [file tp2015125x1.doc]

SUPPLEMENTAL INFORMATION

*Behavioral despair (BD) analysis after adult drug treatment*

ESI adult mice treated with resveratrol or vehicle from PD70-80, underwent FST twice: before (PD69, *pre*) and after (PD 82, *post*; Supplementary Fig. S4) drug treatment. Behavioral despair in the FST was measured in control adult mice (PD82) treated with selisistat or vehicle from PD70-80. Behavioral parameters were measured as described in the main text.

*Locomotor activity (LA) analysis*

Locomotor activity was assessed in adulthood (PD81) in ESI mice treated with Resveratrol from PD14-25 as well as in control mice treated with selisistat from PD70-80 (Supplementary Fig. S5). The apparatus used was composed by five gray opaque plexiglas chambers (20 × 10 cm). Individual mice were introduced into each chamber and their locomotor activity was recorded for 60 min. Behavioral data were collected and analyzed by the ‘EthoVision’ fully automated video tracking system (Noldus, The Netherlands). The “distance moved” (cm) parameter was used to estimate the locomotor activity.

*Statistics*

LA and BD data obtained were initially checked for homogeneity of variance, with measures failing Levene’s test analysed by non-parametric Mann–Whitney procedures. All other parameters were subjected to parametric either student's t test or repeated-measure analysis of variance (ANOVA). ANOVA was followed, in cases of significance (P < 0.05), by post-hoc comparisons using Duncan’s test

# SUPPLEMENTARY TABLE LEGEND

**Supplementary Table S1.** Genes investigated by Quantitative Real time RT-PCR (gene name) and used primers (Applied Biosystems, Branchburg, NJ).

**Supplementary Table S2.** Demographic and clinical data for the control subjects and depressed patients .

**Supplementary Table S3** Demographic and clinical data for the two subgroups of patients divided according to the criterion of childhood parental care (PBI).

**SUPPLEMENTARY FIGURE LEGEND**

*Fig. S1.* Control and early social isolated (ESI) mice displayed similar pattern of exploration of the apparatus during the habituation phase in the social interaction test.

*Fig. S2.* Control and early social isolated (ESI) mice consumed the same amount of water during the sucrose preference test.

*Fig. S3* Frequency (% of observed frequencies) distribution histograms for the immobility measures in the Forced Swimming Test (FST). The distribution of the scores from control mice (blue bars) was narrower (closed to a mean score) compared to ESI (red bars) mice.

*Fig. S4.* (A) Treatment with resveratrol during adulthood did not induce changes in depression-like behavior in ESI mice. Behavioral despair in this experiment was measured before (PRE) and after (POST) the 10 days-drug treatment. (B) Treatment with selisistat during adulthood did not induce changes in depression-like behavior in Control mice. ESI Vehicle, n = 6 (M = 2, F = 4); Resveratrol, n = 6 (M = 2, F = 4); Control: Vehicle, n = 5 (M = 2, F = 3); Selisistat, n = 5 (M = 2, F = 3).

*Fig. S5.* (A) Developmental treatment with Resveratrol did not alter locomotor activity in adult ESI mice. (B) Adult chronic treatment with Selisistat did not alter locomotor activity in Control mice. ESI: Vehicle, n = 4 (M = 2, F = 2); Resveratrol, n = 5 (M = 3, F = 2); Control: Vehicle, n = 6 (M = 2, F = 4); Selisistat, n = 6 (M = 2, F = 4).

*Fig. S6.* Regression analyses revealed no significant correlation between mRNA SIRT1 expression (inverse of ΔCT) and BDI scores in healthy controls.
