# Supplementary material for: Adversity in childhood and depression: linked through SIRT1
Source: Transl Psychiatry. 2015 Sep 1;5(9):e629–. doi: 10.1038/tp.2015.125 (PMC5068813; doi:10.1038/tp.2015.125)
Supplement: Supplementary Table 1 [file tp2015125x2.doc]

**Supplementary Table 1.**

| **Gene Name** | **Code Primer *AB*** | **Specie** |
| --- | --- | --- |
| Sirt1 | Mm00490758_m1 | mouse |
| Sirt2 | Mm01149204_m1 | mouse |
| Sirt3 | Mm00452131_m1 | mouse |
| Sirt4 | Mm01201915_m1 | mouse |
| Sirt5 | Mm00663721_m1 | mouse |
| Sirt6 | Mm00725029_m1 | mouse |
| Sirt7 | Mm01248607_m1 | mouse |
| PGK | Mm01225301_m1 | mouse |
| TBP | Mm00446973_m1 | mouse |
| SIRT1 | Hs01009006_m1 | human |
| TBP | Hs 00427620_m1 | human |
| GUSB | Hs00939627_m1 | human |
