# Supplementary material for: Adversity in childhood and depression: linked through SIRT1
Source: Transl Psychiatry. 2015 Sep 1;5(9):e629–. doi: 10.1038/tp.2015.125 (PMC5068813; doi:10.1038/tp.2015.125)
Supplement: Supplementary Table 2 [file tp2015125x3.doc]

**Supplementary Table 2.**

|  | Controls (N = 19) | Depressed Patients (N = 27) | Statistics |
| --- | --- | --- | --- |
| Age (years) | 47 + 2.0 | 52 + 2.0 | NS |
| Gender ratio (m/f) | 6/13 | 6/21 | Chi-square = 0.72, NS |
| BDI | 4.26 + 0.98 | 23.18 + 2.35 | U = 0.00, P < 0.001, r = 0.83 |

**^#^**Mean + St.err.
