# Supplementary material for: Adversity in childhood and depression: linked through SIRT1
Source: Transl Psychiatry. 2015 Sep 1;5(9):e629–. doi: 10.1038/tp.2015.125 (PMC5068813; doi:10.1038/tp.2015.125)
Supplement: Supplementary Table 3 [file tp2015125x4.doc]

**Supplementary Table 3**

|  | High care (N = 10) | Low care (N = 17) | Statistics |
| --- | --- | --- | --- |
| Age (years) | 54.80 + 2.65 | 50.41 + 3.23 | NS |
| Gender ratio (m/f) | 2/8 | 3/14 | Chi-square, NS |
| BDI | 19.30 + 2.22 | 25.47 + 3.43 | NS |
| HAMD | 11.30 + 1.16 | 13.88 + 1.06 | NS |
| Age of MD first diagnosis | 40.90 + 3.93 | 30.00 + 2.99 | t =2.20, P = 0.04 |

**^#^**Mean + St.err.
