# Supplementary material for: Adversity in childhood and depression: linked through SIRT1
Source: Transl Psychiatry. 2015 Sep 1;5(9):e629–. doi: 10.1038/tp.2015.125 (PMC5068813; doi:10.1038/tp.2015.125)
Supplement: Supplementary Figure 1 [file tp2015125x5.ppt]

## Slide 1
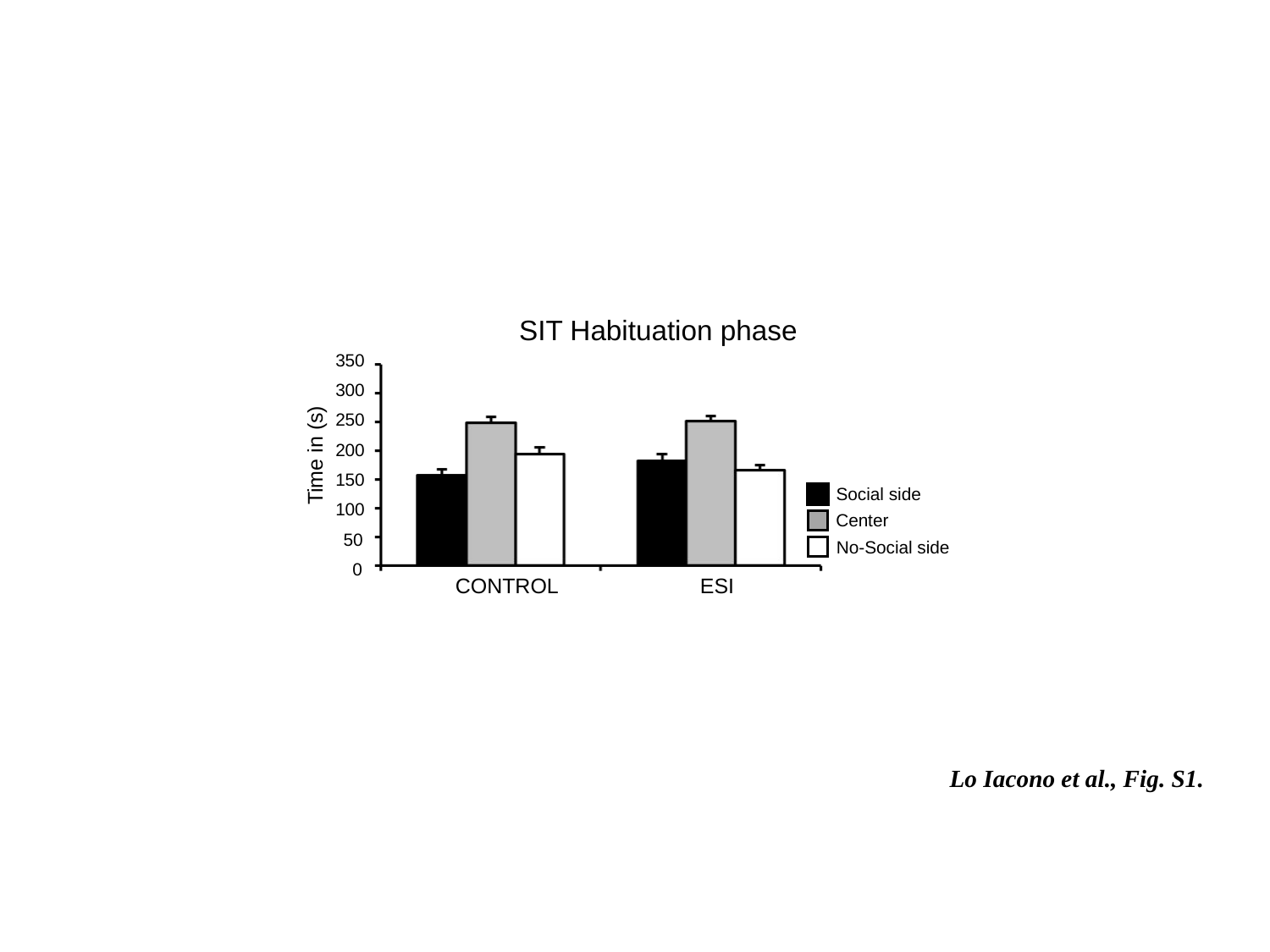

SIT Habituation phase
350
300
250
200
Time in (s)
150
Social side
100
Center
50
No-Social side
0
CONTROL
ESI
Lo Iacono et al., Fig. S1.
