# Supplementary figures and images for: Adversity in childhood and depression: linked through SIRT1
Source: Transl Psychiatry. 2015 Sep 1;5(9):e629–. doi: 10.1038/tp.2015.125 (PMC5068813; doi:10.1038/tp.2015.125)

## Slide 1
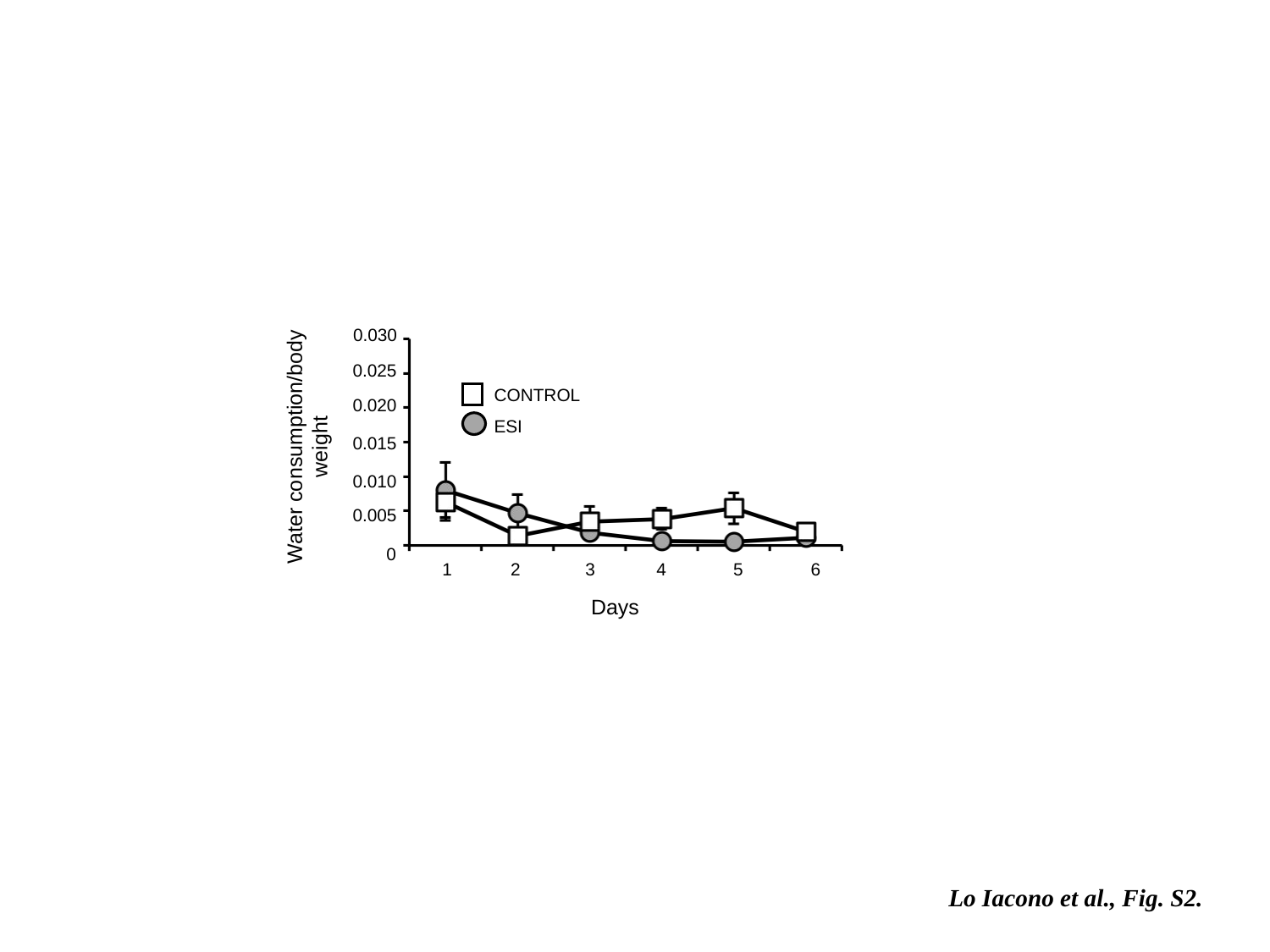

0.030
0.025
CONTROL
0.020
ESI
Water consumption/body weight
0.015
0.010
0.005
0
1
2
3
4
5
6
Days
Lo Iacono et al., Fig. S2.

Supplement: Supplementary Figure 2 [file tp2015125x6.ppt]

## Slide 1
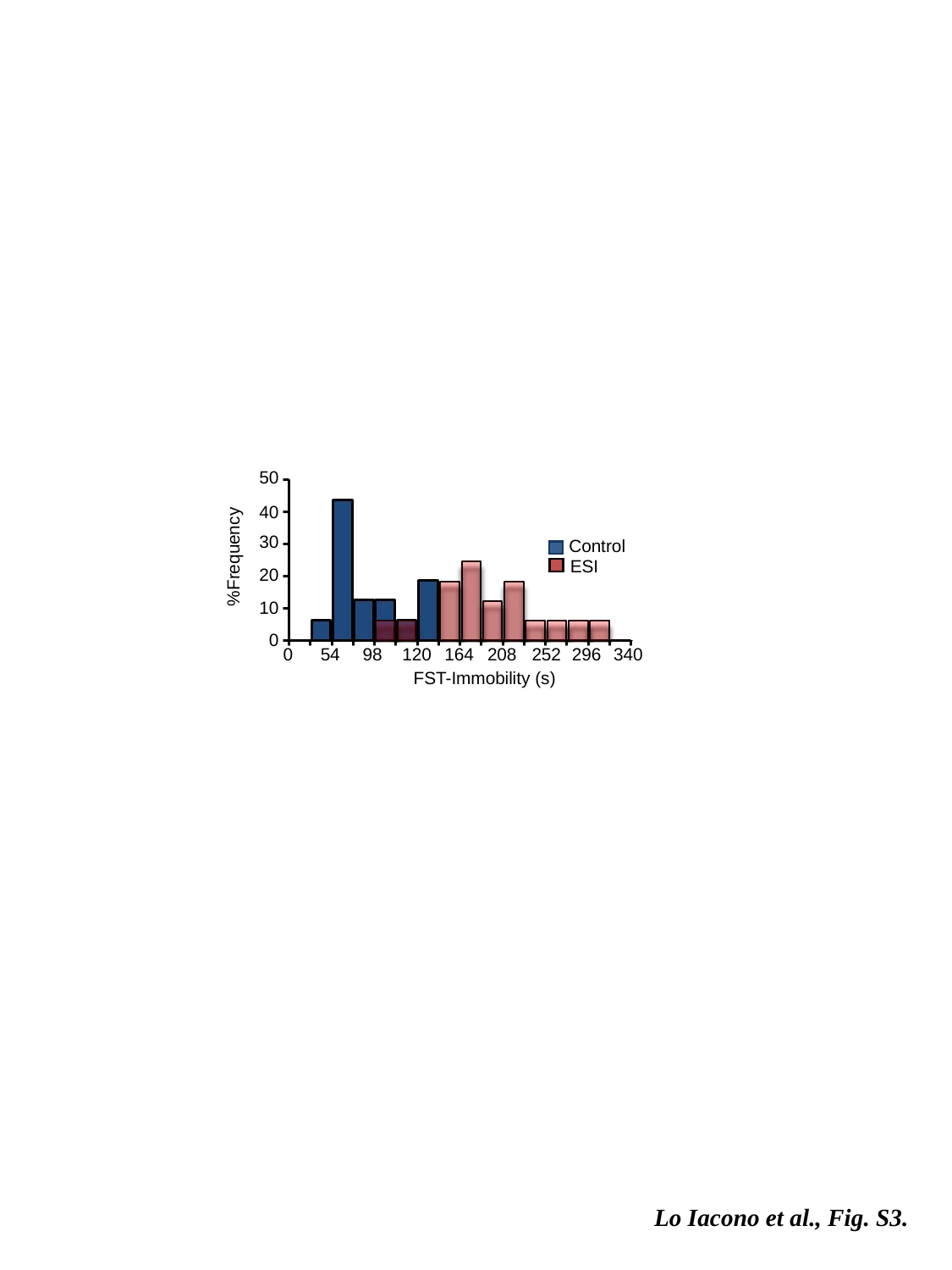

50
40
30
Control
%Frequency
ESI
20
10
0
0
54
98
120
164
208
252
296
340
FST-Immobility (s)
Lo Iacono et al., Fig. S3.

Supplement: Supplementary Figure 3 [file tp2015125x7.ppt]

## Slide 1
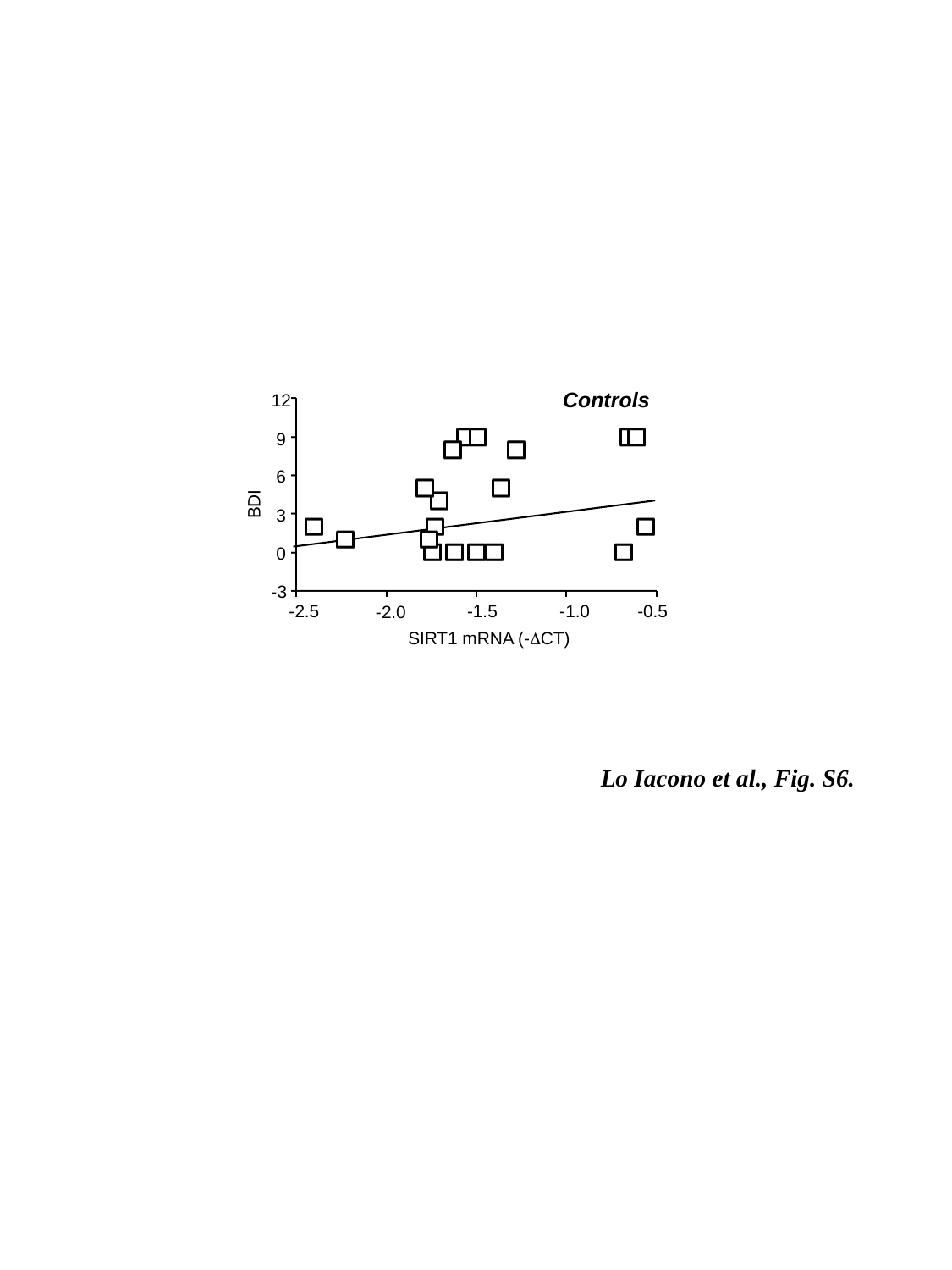

Controls
12
9
6
BDI
3
0
-3
-2.5
-1.5
-1.0
-0.5
-2.0
SIRT1 mRNA (-ΔCT)
Lo Iacono et al., Fig. S6.

Supplement: Supplementary Figure 6 [file tp2015125x10.ppt]
