# Supplementary material for: Adversity in childhood and depression: linked through SIRT1
Source: Transl Psychiatry. 2015 Sep 1;5(9):e629–. doi: 10.1038/tp.2015.125 (PMC5068813; doi:10.1038/tp.2015.125)
Supplement: Supplementary Figure 4 [file tp2015125x8.ppt]

## Slide 1
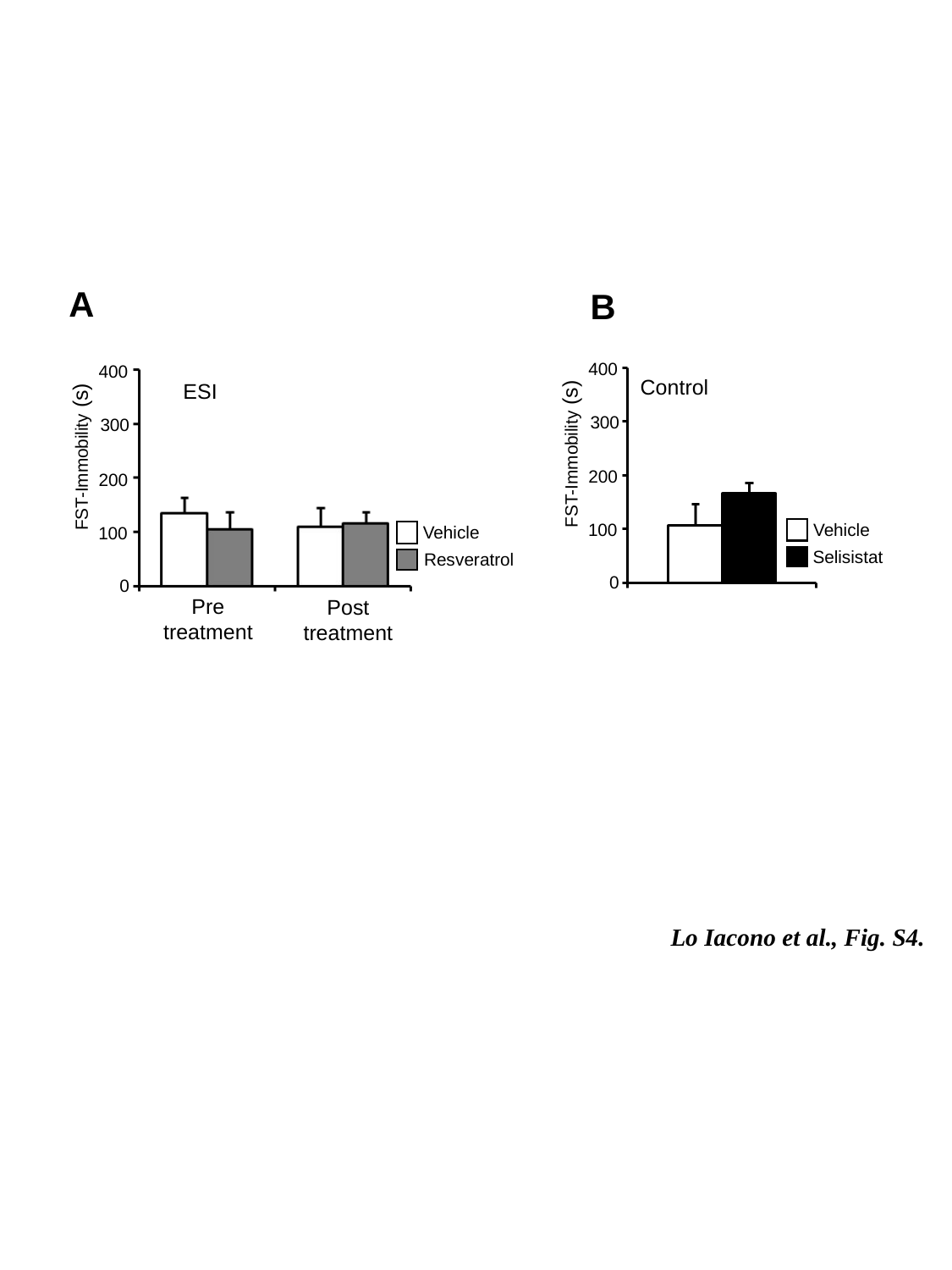

A
B
400
Control
300
FST-Immobility (s)
200
100
Vehicle
Selisistat
0
400
ESI
300
FST-Immobility (s)
200
Vehicle
100
Resveratrol
0
Pre treatment
Post
treatment
Lo Iacono et al., Fig. S4.
