# Supplementary material for: Adversity in childhood and depression: linked through SIRT1
Source: Transl Psychiatry. 2015 Sep 1;5(9):e629–. doi: 10.1038/tp.2015.125 (PMC5068813; doi:10.1038/tp.2015.125)
Supplement: Supplementary Figure 5 [file tp2015125x9.ppt]

## Slide 1
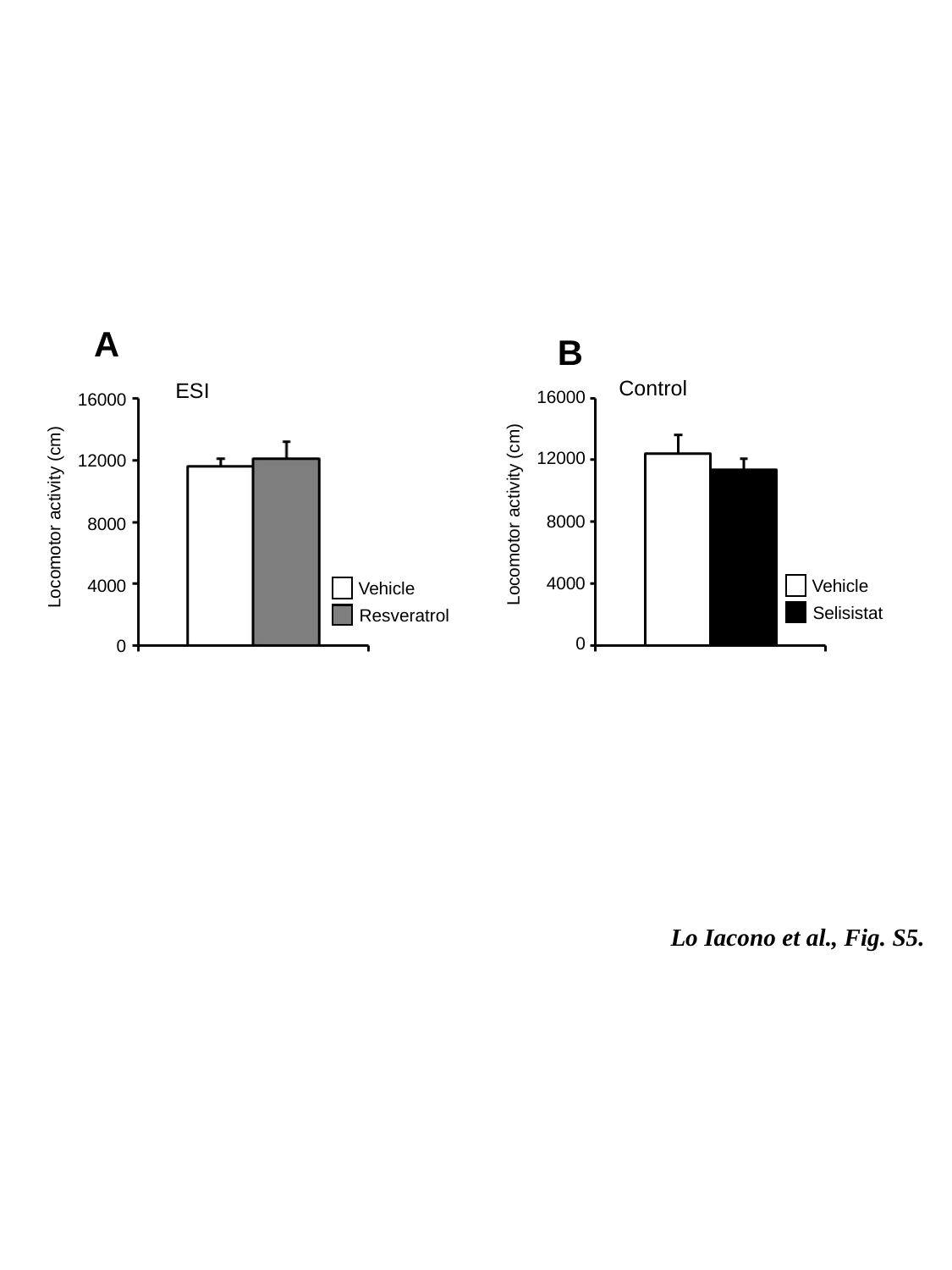

A
B
Control
16000
12000
Locomotor activity (cm)
8000
4000
Vehicle
Selisistat
0
ESI
16000
12000
Locomotor activity (cm)
8000
4000
Vehicle
Resveratrol
0
Lo Iacono et al., Fig. S5.
